# Towards the Development and Verification of a 3D-Based Advanced Optimized Farm Machinery Trajectory Algorithm

**DOI:** 10.3390/s21092980

**Published:** 2021-04-23

**Authors:** Tomáš Řezník, Lukáš Herman, Martina Klocová, Filip Leitner, Tomáš Pavelka, Šimon Leitgeb, Kateřina Trojanová, Radim Štampach, Dimitrios Moshou, Abdul M. Mouazen, Thomas K. Alexandridis, Jakub Hrádek, Vojtěch Lukas, Petr Širůček

**Affiliations:** 1 Department of Geography, Faculty of Science, Masaryk University, 611 37 Brno, Czech Republic; leitnerfilip@gmail.com (F.L.); pavelka93@gmail.com (T.P.); leitgeb.simon@gmail.com (Š.L.); ktrojanova@mail.muni.cz (K.T.); r.stampach@centrum.cz (R.Š.); j.hradek77@gmail.com (J.H.); 2 Agricultural Engineering Laboratory, Faculty of Agriculture, Aristotle University of Thessaloniki, 54124 Thessaloniki, Greece; dmoshou@agro.auth.gr; 3 Precision Soil and Crop Engineering (SiTeMan), Faculty of Bioscience Engineering, Ghent University, 9000 Gent, Belgium; abdul.mouazen@ugent.be; 4 Laboratory of Remote Sensing, Spectroscopy and GIS, Faculty of Agriculture, Aristotle University of Thessaloniki, 54124 Thessaloniki, Greece; thalex@agro.auth.gr; 5 Department of Agrosystems and Bioclimatology, Faculty of Agronomy, Mendel University, 613 00 Brno, Czech Republic; vojtech.lukas@mendelu.cz (V.L.); qqsiruce@node.mendelu.cz (P.Š.)

**Keywords:** controlled traffic farming, coverage path planning, digital elevation model, mission planning, soil compaction

## Abstract

Efforts related to minimizing the environmental burden caused by agricultural activities and increasing economic efficiency are key contemporary drivers in the precision agriculture domain. Controlled Traffic Farming (CTF) techniques are being applied against soil compaction creation, using the on-line optimization of trajectory planning for soil-sensitive field operations. The research presented in this paper aims at a proof-of-concept solution with respect to optimizing farm machinery trajectories in order to minimize the environmental burden and increase economic efficiency. As such, it further advances existing CTF solutions by including (1) efficient plot divisions in 3D, (2) the optimization of entry and exit points of both plot and plot segments, (3) the employment of more machines in parallel and (4) obstacles in a farm machinery trajectory. The developed algorithm is expressed in terms of unified modeling language (UML) activity diagrams as well as pseudo-code. Results were visualized in 2D and 3D to demonstrate terrain impact. Verifications were conducted at a fully operational commercial farm (Rostěnice, the Czech Republic) against second-by-second sensor measurements of real farm machinery trajectories.

## 1. Introduction

Decreasing soil health causes, among other issues, a barrier to producing more high-quality food and has become a widespread challenge across the world. The importance of soil is evident as it is one of the essential factors underpinning the survival of all living things. For that reason, issues related to soil health and soil productivity are defined in countless legally binding documents, scientific papers, strategies, best practices, models, and applications, etc. (e.g., Feiden et al. [1]). The majority of (developed) countries address soil productivity and health—in particular, issues relating to soil erosion and soil compaction—on a legislative basis.

The challenge of optimizing movement on agricultural soil was first addressed before the advent of mechanization [2]. At that time, the time-consuming nature of cultivating fields and minimizing the load on agricultural animals was an issue; nowadays, interest has shifted to optimizing the movement of agricultural machinery to reduce both the environmental burden and operating costs while increasing efficiency [3,4]. Costs are defined in terms of materials, finances, and time [5]. The most tangible savings include reductions in fuel, time, and human resources [6,7,8]. In addition, reducing the requirements for water for irrigation, fertilizer, and pest control helps to minimize adverse effects on the environment such as soil compaction [9] or/and erosion [7,10]. Plot identification was defined by Reznik et al. [11], while the visualization of farm trajectories was presented by Reznik et al. [12] and Charvat et al. [13].

Controlled traffic farming (CTF) is a strategy to minimize soil erosion and compaction, which is being implemented worldwide. CTF systems use a modular working width and traffic gauge equipment together with precise guidance to constrain all load-bearing traffic to permanent lanes [14]. CTF and its impact on soil quality have been addressed intensively during the last two decades: Noguchi and Terao [15], Yan et al. [16], Meuth and Wunsch [17], Oksanen and Visala [5], Driscoll [6], Jin and Tang [10,18], Gao et al. [19], Gutman and Ioslovich [20], Hameed et al. [21], Biglarbegian and Al-Turjman [3], Hameed [7], Chyba et al. [9], Plessen and Bemporad [22,23], Rodias et al. [8], Kroulík et al. [24], and Tu and Tang [25]. Route planning seems an essential requirement for successful CTF implementation. Related papers mostly deal with strictly limited route planning approaches, such as turns by Jin and Tang [18] or the split-and-merge approach by Oksanen and Visala [5]. In contrast, Jin and Tang [10], Hameed [7] and Hameed et al. [21] aimed at the optimization of farm machinery trajectories in a more complex way. Only Jin and Tang [10] and Hameed [7] also take elevation into account when optimizing trajectories.

With respect to the work performed so far, the following issues concerning CTF remain open:

- Plot segmentation;
- As a variable, among others, according to a given time;
- With respect to a desired number of machine sequences;
- With respect to elevation (slope);
  ○ Optimization of the entry and exit points of a plot segment;
  ○ The modeling of trajectories based on real obstacles;
  ○ The return from a (finished) plot segment to a place that in reality allows the machine to truly leave the plot;
- A formalized workflow that addresses the optimization of farm machinery trajectories in a complex manner.

The main goal of this study was to develop a proof-of-concept algorithm for optimizing farm machinery trajectories. The chief motivation behind the presented research was to reduce the risk of creating soil compaction by means of on-line optimization and logistical algorithms of trajectory planning for soil-sensitive field operations, taking into account different configurations of machinery. Fuel reductions and the saving of time as well as of human and financial resources (e.g., lowering machinery wear) are economic side-benefits of the chosen approach. The general main objective was further developed into the following specific research questions:

1. What are the input requirements for designing a CTF-based algorithm for optimizing farm machinery trajectories?
2. What are the ways of formalizing the identified requirements as a basis for their automatization?
3. How does the (theoretically) optimized trajectory of a farm machine differ from the reference (real) trajectory of such a machine?

The following terminology is used consistently throughout the paper. The term “plot” is used at the conceptual level, primarily for modeling, while the term “field” is related to the application level, e.g., when talking about harvest.

This paper is structured as follows:

- Section 2 analyzes state-of-the-art approaches related to the optimization of farm machinery trajectories.
- Section 3 describes the methodology developed within this paper when building on existing approaches; accordingly, it:
  ○ Introduces data from the fully operational reference farm at Rostěnice, the Czech Republic (Section 3.1);
  ○ Deals with the pre-processing of data from the reference farm in order to homogenize them (Section 3.2);
  ○ Summarizes the functional requirements for optimal trajectories (Section 3.3);
  ○ Presents an open, scalable, and modular proof-of-concept algorithm for optimizing farm machinery trajectories (Section 3.4);
  ○ Evaluates statistical differences between reference (real) trajectories and optimized (modeled) farm machinery trajectories (Section 3.5).
- Section 4 presents results comprising:
  ○ The formalization of the developed algorithm through unified modeling language (UML) activity diagrams (Section 4.1);
  ○ The formalization of the developed algorithm through pseudo-code notation (Section 4.2 and Supplementary Materials);
  ○ Comparison of the (theoretically) optimized and reference (real) trajectories within the study area (Section 4.3).
- Section 5 presents the discussion of the achieved results, including their confrontation with existing approaches in the following respects:
  ○ Fulfilment of the defined requirements for designing a CTF-based algorithm (Section 5.1);
  ○ Ways of formalizing the developed algorithm (Section 5.2);
  ○ In-depth differences between the (theoretically) optimized and the reference (real) trajectories (Section 5.3).

## 2. Related Work

Optimizing farm machinery trajectories with respect to CTF in order to minimize environmental impacts is a very complex task which can be viewed from different angles. Essentially, all analyzed studies focused mainly on partial aspects of achieving this task.

The basic pre-condition for planning the optimal trajectory of an agricultural machine is to ensure that the machine passes the entire plot, e.g., it harvests the entire crop [17]. The optimal trajectory is considered to be the shortest trajectory through all the specified points in the search area, in our case plot, which relates to the so-called Travelling Salesman Problem (TSP) [17]. A heuristic algorithm which can be used to solve this problem is the Lin–Kernighan (LK) method. LK is an iterative method that begins with a random inspection and performs optimization in pairs according to the steps required to reach local minima [17]. The LK method is computationally demanding and, among other things, unsuitable for real-time use. The LK method can, however, be enriched with a genetic element called Evolutionary Lin–Kernighan (ELK). This enhancement is based on genetic operators with similar architectures [17]. The quality of the trajectory according to the ELK algorithm was compared with the quality according to the original LK algorithm [17]. The ELK algorithm provided better results, creating paths of higher quality in fewer iterations. Among others, the test tours created by the ELK algorithm were evaluated significantly faster, because the LK method needed to make several more changes due to the pre-optimized sections of the sub-trajectories.

Planning optimal trajectories for plots that have complex shapes or contain obstacles is a different level of application than (E)LK. An algorithm based on the limited Delaunay triangulation [16] or a complex approach based on the generation of field-work tracks, the clustering of these tracks into blocks, the generation of headland paths, and a genetic algorithm to optimize the sequences [7,21] can be used for this purpose. The principle of dividing a complex-shaped plot into smaller objects that can be cultivated separately is the main idea of the study by Oksanen and Visala [5]. Such a method is referred to as SPLIT-AND-MERGE. It aims to identify the “best” predominant direction for the machine trajectory for the defined plot fragments. The processing capacity of agricultural machinery, such as field harvesters, is included in this calculation. Once the field is divided into sub-shapes of simple form, which are convex or almost convex, the optimal solution for field control can be found using the longest edge [5].

**Figure 1:**
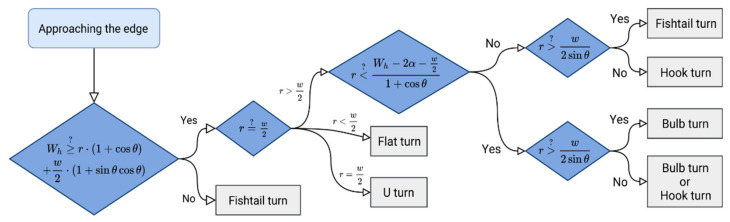
Decision tree for selecting the most suitable type of rotation. Meanings of variables are as follows: *r*—radius of rotation of the vehicle, *w*—working width, *W_h_*—headland width, *θ*—swath direction, α is the angle of arch, which is a function of *r*, *θ*, and *w*. Adopted from: Jin and Tang [18].

Setting the “best” predominant direction of the trajectory is another aspect of optimal CTF-based trajectory planning. Jin and Tang [18] consider the “best” direction of the trajectory to be the most important aspect. A trajectory composed of straight parallel passages with alternating directions is the simplest general strategy for operating in agricultural locations, as such passages can be easily followed by agricultural machinery. More specifically, the simplest method identified by Jin and Tang [18] follows the longest edge of a plot. However, the optimized trajectory could only be obtained for plots with a simple convex shape (ideally, a rectangle). It is essential to take into account field boundary irregularities in order to achieve a general coverage planning solution. In order to find the optimal solution, coverage path planning requires decomposition and directional search algorithms to minimize the cost of the function of angular rotations [8]. An accurate estimate of turning costs is also an important aspect. Due to the limited minimum radius of the agricultural machinery, the predefined row width, and the limited headland space, “U”-shaped rotation may not be used in some situations. Instead, other types of rotation can be used, such as “flat turn” or “light bulb” (more types are listed in Figure 1). Algorithm verification showed that turning costs can be reduced by up to 16%, and, at the same time, no tested machine performed worse than when following the original travel plan [18]. The issue of turning and its optimization was further investigated using unmanned technologies [25].

When optimizing a trajectory, processing can lead to sharp paths to achieve field coverage [23]. This requires the smoothing of the trajectory into a machine-feasible form. This part of optimization can employ three types of post-processing adjustment: circular segments, generalized basic paths, and bi-elemental paths [23]. The third adjustment method eliminates the time dependence and replaces it with the spatial representation of the model, as in Gao et al. [19], Plessen and Bemporad [22,23].

Advanced CTF-based computational methods are as follows. A combination of a neural network and a genetic algorithm was introduced by Noguchi and Terao [15] when processing optimized movement on agricultural land by autonomous vehicles (agricultural robots). Unlike the movement of agricultural vehicles, which use larger steering angles, such autonomous movement exhibits high nonlinearity [15]. Gutman and Ioslovich [20] also proposed an algorithm for the control of autonomous agricultural vehicles based on the optimal allocation of working time and trajectory planning for agricultural activities.

As far as the authors are aware, no publicly available complex CTF-based algorithm for optimizing farm machinery trajectories exists that takes into account the following: variable plot segmentation including the optimization of its entry and exit points and the modeling of reality-based obstacles. The approaches identified above represent partly isolated solutions, the most complex research represented by Hameed [7], Hameed et al. [21], and Jin and Tang [10].

## 3. Materials and Methods

This section presents the basic components and methodology commensurate with the main goal of the paper—specifically, to develop a proof-of-concept algorithm for optimizing farm machinery trajectories.

### 3.1. Reference (Real) Farm Machinery Trajectories

**Figure 2:**
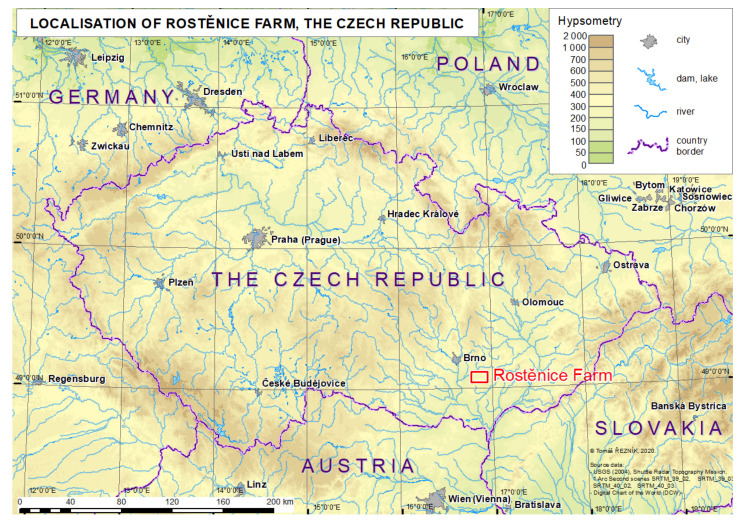
Geographical location of the Rostěnice Farm.

**Figure 3:**
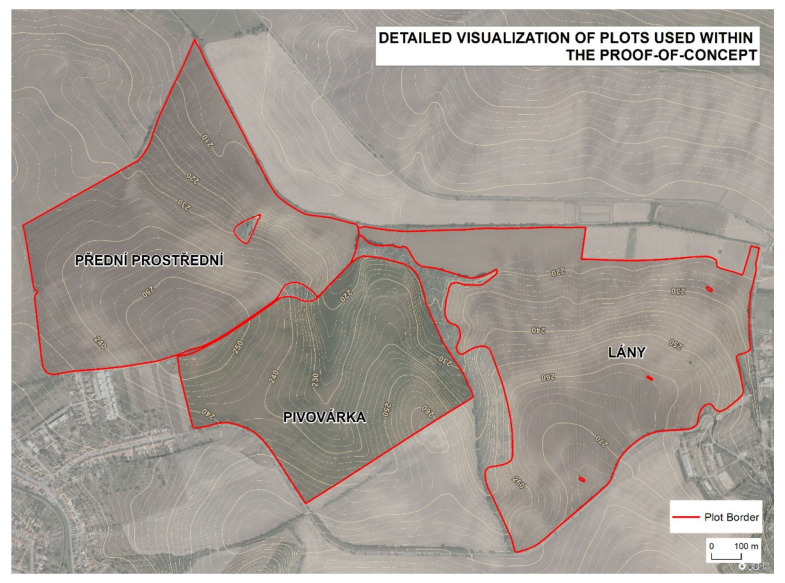
Detailed visualization of plots used within the proof-of-concept.

Reference data acquisition was conducted at the Rostěnice cooperative farm in the south-eastern part of the Czech Republic (Figure 2). Data were measured by a CASE IH AXIAL FLOW 9120 (Case IH., Kunde, Germany) field harvester equipped with an AFS Pro 700 (Case IH., Kunde, Germany) monitoring unit for three fields: Lány, Pivovárka, and Přední Prostřední (Figure 3). Reference (real) farm machinery trajectories were in WGS84 (World Geodetic System) as defined in the proj4 library [26]. The EPSG (European Petroleum Survey Group) code of the used coordinate reference system was “4326” [27]. The measurements were of GNSS-RTK quality (Global Navigation System of Systems—Real Time Kinematics), i.e., they provided a spatial resolution of less than 0.1 m [28]. Measurements were taken continuously each second at an average speed of 1.55 m×s^−1^, recommended as optimal at the Rostěnice Farm for cereal harvesting by the CASE IH AXIAL FLOW 9120 field harvester (for more details, see Řezník et al. [29]). Reference data also contained vertical information; however, such vertical information was not used during the conducted experiment due to the following:

- First, the main motivation was to homogenize input elevation information to both reference (real) as well as optimized (modeled) farm machinery trajectories. Elevation was computed from the Digital Terrain Model (DTM) 5th Generation (hereinafter DTM 5G) provided by the Czech national mapping organization, the Czech Office for Survey, Mapping and Cadastre. DTM 5G has positional accuracy equal to 0.14 m and vertical accuracy equal to 0.18 m.
- Second, elevation was not available for all the (real) reference farm machinery trajectories. Typically, headlands (turns) have gaps in the measured data [29].

Note, reference (real) farm machinery trajectories were available for three years for each plot. Trajectories differed only partially in headlands. As such, this paper presents only one real farm machinery trajectory for one year to increase the clarity of (mainly) maps.

### 3.2. Pre-Processing of Reference (Real) Farm Machinery Trajectories

**Figure 4:**
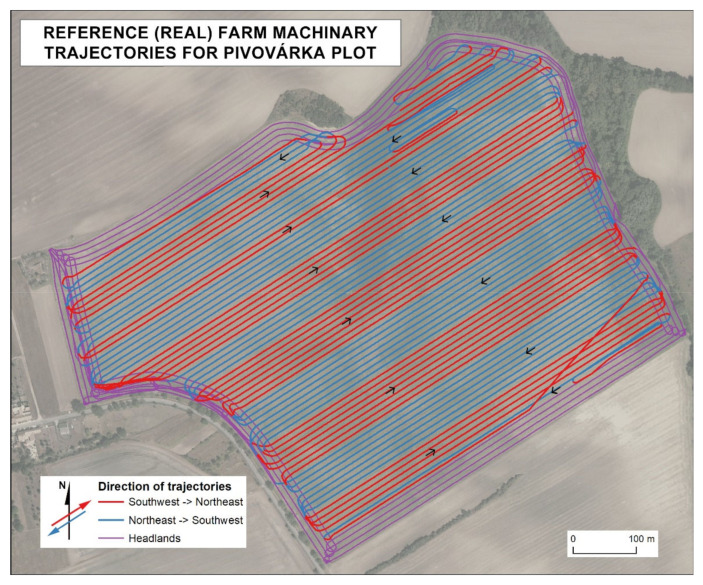
Reference (real) farm machinery trajectories with marked direction.

Reference (real) farm machinery trajectories were based on the sequence of point data obtained by harvesters delineating a continuous trajectory line (Table 1). While modeling trajectories in areas with reference data gaps (mostly turns), orthophoto images as well as continuity, the diameters of turns, and the geometry of the plot were taken into account for the reconstruction of possible trajectories (Figure 4). The origin of gaps in reference (real) point data is elaborated in Řezník et al. [29]. The order of measurements was maintained. Reference (real) farm machinery trajectories were mostly incomplete in the headlands. A constant trajectory radius of U-turn was used to add the missing parts homogeneously. The request to harvest 100% of the production was also met. Such a requirement falls under the coverage path planning [6,21]. The development of an algorithm for the automatization of pre-processing was not considered since harvesters follow various specific harvest requirements. Such specific harvest requirements lead in some cases to unexpected trajectories. Therefore, the automatized reconstruction of reference (real) trajectories is not entirely possible when larger amounts of data are missing.

### 3.3. Functional Specification and Its Verification

The functional requirements were as follows, these originating from (1) Section 2, (2) exploratory analysis of trajectories within and beyond the reference farm [12,13,29,30], and (3) consultations with agronomists at Rostěnice Farm for the development of an optimized (modeled) farm machinery trajectory that is applicable to plant production on the fields:

- Efficient divisions of plots:
  ○ Field fragmentation in line with its shape [5,6,21];
  ○ Field fragmentation in line with terrain characteristics (not addressed by any discovered paper).
- Production of the whole harvest: 100% of the cultivated crop needed to be harvested [6,17].
- Avoidance of long-term obstacles: long-term obstacles in the field needed to be addressed automatically [21]. Long term in this context means the existence of an obstacle for more than one agronomic season. Note, short-term obstacles were not taken into account.
- Minimization of overall trajectory length: trajectory length should be kept at a minimum as it affects fuel consumption, CO_2_ emissions, and operating time (e.g., Hameed [7]; Rodias et al. [8]; Kroulík et al. [24]).
- Minimization of the number of passes: reduction in soil compaction [9,14].
- Selection of appropriate U-turn shape: in line with research developed by Jin and Tang [18].
- Adaptability to relevant farm machinery (such as vehicle width, axle load, tire pressure) [31,32,33].
- Analysis of terrain impacts: as the minimization of elevation gain is an expected a priori impact of trajectory optimization, with further impacts as follows:
  ○ Steepness of the slopes as a key input to address erosion [7,10];
  ○ Selection of field entry/exit points according to their altitude (not addressed by any discovered paper).

The requirements were transformed into stand-alone functions of the developed proof-of-concept algorithm, as presented in Section 3.4.

### 3.4. Development of Optimized (Modeled) Farm Machinery Trajectory Algorithm

The methodology used in this study followed the “waterfall approach”, as described by Royce [34]. The methodology of optimizing farm machinery trajectories was designed as a proof-of-concept generally applicable to any plot(s). As such, the methodology consists of modules in order to provide customizable solutions reflecting the needs of users. The experiment presented in this paper is specifically focused on field-harvesting operations.

Note the nomenclature used within both the description of the methodology below as well as in the UML diagrams presented in Section 4.1. of Supplementary Materials. The name of each module is presented in capitalized stand-alone words such as “*Plot fragmentation*”. The name of a diagram which at the same time does not represent a module is depicted as a pascal snake case, separated by underscores, such as “*Calculate_Ideal_Move_Vector*”. The name of a function is presented in snake case notation such as “*get_season*”.

**Figure 5:**
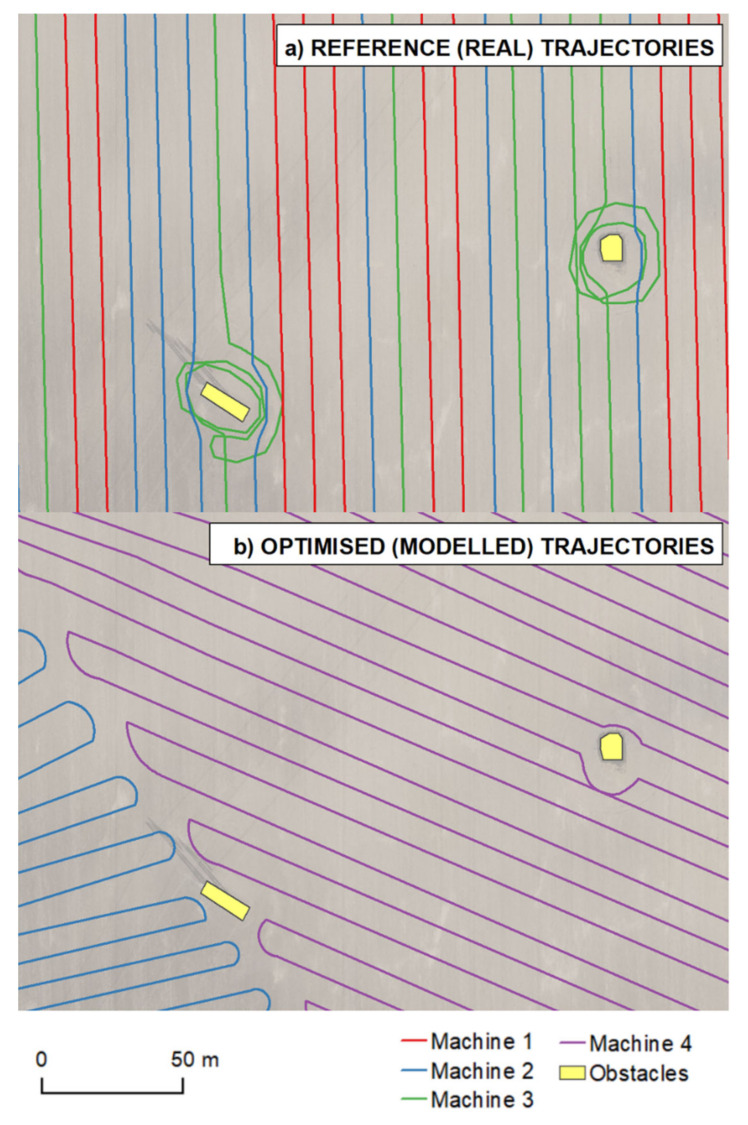
Examples of obstacle avoidance in reference (real) trajectories (**a**) and optimal trajectories (**b**). Note that the left obstacle in the left image lies on the border of trajectories in the right image.

The proof-of-concept workflow comprises the following modules:

- *Data import*: the following kinds of data are required as a prerequisite for running the presented algorithm optimizing a farm machinery trajectory:
  - DTM with its spatial accuracy (SA) in line with Equation (1):
    (1) $$Min_{SA}\;\geq \frac{SL_{SPAN}}{2}$$
    where:
    ○ *Min_SA_* corresponds to the minimal spatial accuracy of an imported DTM;
    ○ *SL_SPAN_* corresponds to the (desired) machine operating on a plot (for details, see module B).
  - Geometry of plots with spatial accuracy consistent with the DTM, i.e., in line with Equation (1).
  - Obstacles in terms of objects that have a size greater than the maneuvering capabilities of a machine performing the desired field operation. Typical examples are trees, bushes, and ponds, separately or in a form of green and blue infrastructures [35], or human-made objects such as power poles.
- *Parameter settings:*
  - Span length of a (desired) machine operating on a plot. For example, the span length equals the width of the cutting bar of a harvester. Span length may, therefore, vary for the identical machine conducting various field operations such as sowing or harvesting. Experiments described within this paper narrowed the focus only to the field-harvesting operation. Only cereals (winter wheat, spring barley, corn) were harvested in the proof-of-concept fields. The span length within the proof-of-concept conducted in this study was 9.15 m for the Pivovárka plot and 10.4 m for Lány and Přední Prostřední plots. Such values originated from the existing machinery at Rostěnice Farm and were kept to allow the comparability of results between the computed optimized (modeled) route of a farm machine and the reference (real) farm machinery trajectory.
  - Machine length of a (desired) machine operating on a plot. Additionally, in this case, machine length varied between various field operations. The machine length within the proof-of-concept conducted in this study was 7 m for all the plots. Such a value originated from the existing machinery at Rostěnice Farm and was used for the same reason indicated in point B. a.
- *Plot fragmentation*: such a module aims at simplifying the plots by splitting them into smaller fragments [5]. In these sections, it is easier to calculate a trajectory that will be less costly. Fragmentation into plot segments is designed to allow the employment of more machines.
  - *Division_Based_On_Vertices*—calculates the angle for every three consecutive vertices. The function is supposed to split the corresponding plot into segments when an angle is found to be greater than 180°. Such an output is achieved by driving a perpendicular from the apex of an angle to the opposite field boundary. The rationale follows the goal of preventing the existence of inaccessible segments that would otherwise have to be managed separately.
  - *Division_Based_On_Aspect*—a plot is split with respect to the aspect function, calculated from the input DTM. In such a case, crossing the slope is prevented. The natural structure of the terrain can be observed thanks to this division.
  - *Division_Based_On_Slope*—detects uncultivable segments of the plots. If the slope in any part of the plot reaches 10° or more, it will be considered an obstacle. Such a sub-module prevents running over clods and possibly damaging the machine.
- *Trajectory calculation*: for each plot, subdivision is a core functionality of the whole algorithm. It consists of three sub-modules, each representing a different phase of the presented process.
  - *Initiation*—identifies the plot division starting point along with the optimized (modeled) route. It comprises the following:
    - *Create_headlands* creates a buffer area around the plot segment boundary with a width of one machine length, a constraint determining the minimum space required for successful rotation.
    - *Get_starting_point*—the computation goes as follows in the case of there being no predefined entry point. The position of a starting point is determined by finding a boundary vertex which is in the closest proximity to the spot of highest elevation.
    - *Calculate_Ideal_Move_Vector* determines the initial movement direction of the machine based on the position of the starting point. In general, two resulting directions are possible: (1) parallel with the plot segment boundary or (2) following a contour line. The first case is the preferred one when deviation from a contour line is no more than 5°.
  - *Movement* prolongs a trajectory by adding new vertices in the direction determined by the previous phase. Such a sub-module also performs an assessment of the turn necessity as follows:
    - *Follow_direction_or_boundary* (until the buffer as defined in the D. a. i. point is crossed).
  - *Rotation*—once the previous sub-module “*Movement*” evaluates that the machinery is supposed to make a turn, this sub-module evaluates possibilities and picks the most suitable rotation type to perform [18]. In the case when a rotation is not feasible, the current position is considered as the end vertex. After a successful rotation, another iteration of the “*Movement*” sub-module is initiated.
- *Bypass the obstacles*: such a module performs a modification of the first iteration of the created trajectory, which has not yet taken any obstacle objects into consideration [18]. The presented proof-of-concept approach comprises reviews of the suggested trajectory vertex’s position with obstacles buffered by one half of the width of the cutting bar. An alternative route along the obstacle is then calculated for a route segment in between so-called touch vertices (vertices representing the first and last valid vertex according to the corresponding obstacle). The approach of Jin and Tang [18] was adopted as there are two route types available at each time: “left” and “right”. A smaller underlying area results from the division of the obstacle made by the original trajectory (Figure 5).
- *Return to an exit point*: a module through which it is possible to leave the plot in reality. In our study, the entry/exit points were identified in line with reference data, reference (real) trajectories, DTM 5G and orthophotos. The entry/exit points were then confirmed by agronomists at Rostěnice Farm.
- *Export*: Exporting capabilities were defined to obtain the resulting optimized (modeled) routes for farm machinery in an exchange format. The exported results were then used in statistical analyses as well as in proof-of-concept visualizations. The “*Export*” module comprises the following sub-modules:
  - Export trajectory as geographical file format (e.g., Shapefile or GeoJSON).
  - Export ENTER and END points as geographical file format (e.g., Shapefile or GeoJSON).

### 3.5. Statistical Evaluation

The resulting optimized (modeled) trajectories were verified against second-by-second sensor measurements of reference (real) farm machinery trajectories. Comparison of trajectories was based on the following descriptive statistics measures calculated for both types of trajectories, reference and modeled. Length and elevation were computed on the basis of the coordinate reference system Universal Transverse Mercator (UTM) zone 33N [36]. The Baltic Height System was used when computing elevation tasks as it is the default vertical system of DTM 5G reference data.

The following measures were used to compare reference (real) trajectories on the one hand and optimized (modeled) trajectories on the other:

(a) Trajectory length in meters with computations based on the coordinate reference system UTM zone 33N for both (real) reference farm machinery trajectories and optimized (modeled) trajectories.
(b) The number of turns needed for machinery to harvest a whole field including its subdivisions. In the case of (real) reference trajectories, the changes in a path’s direction caused by movement along the plot border were not considered as turns.
(c) Elevation gain calculation was based on values derived from DTM 5G reference data resampled to a one meter step generated along the trajectory. The final value of elevation gain for the given trajectory represents the sum of the positive differences for the “*next_value-current_value*” variable.

## 4. Results

**Figure 6:**
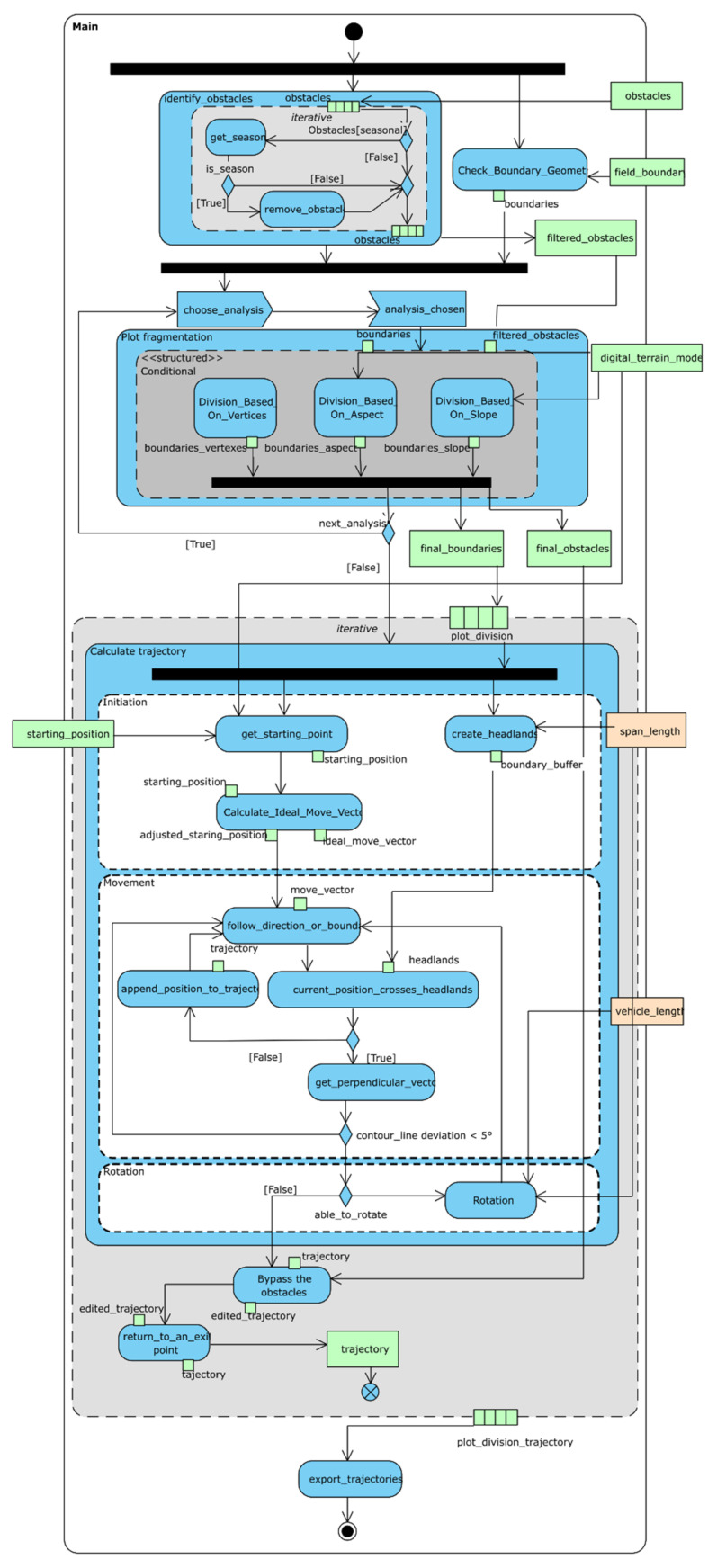
Overall unified modeling language (UML) activity diagram for an optimized (modeled) farm machinery route algorithm.

Five main results were achieved during the development of the optimal farm machinery route algorithm:

- UML activity diagrams documenting the developed algorithm (see the overall level of detail in Figure 6 as an example, and six remaining UML diagrams in Supplementary Materials);
- Pseudo-code of the developed algorithm (in Supplementary Materials);
- Trajectories created according to the developed algorithm (since the used procedure is designed as parameterizable and scalable, there are several trajectory variants, as depicted in Supplementary Materials);
- Comparison of measures calculated from both types (real and theoretical) of trajectories (trajectory lengths, number of turns, and elevation gain; see Table 2 and Supplementary Materials);
- 2D and 3D cartographic outputs (throughout the remaining four kinds of results).

### 4.1. Documentation of the Developed Algorithm in Unified Modeling Language (UML)

The methodology of optimizing farm machinery routes presented in Section 3.4 is designed to be generally applicable to any field. As such, the methodology consists of modules in order to provide customizable solutions. The developed algorithm is documented by means of eight UML process diagrams, as depicted in Figure 6 (an overall diagram) and in Supplementary Materials (the remaining six detailed UML diagrams).

So-called UML Activity Parameter Nodes in green and light-yellow color were used to express the data and parameter inputs. Both (data and parameter inputs) are understood as a prerequisite to run the presented algorithm. Green Object nodes were used to represent data flow within the process. Modules as well as functions and subdiagrams are depicted as blue UML Activity or Action nodes. The *Parameter Settings* module defines input parameters such as the span length of a machine operating on a field. The *Plot Fragmentation* module simplifies the field geometry by splitting it into smaller fragments where three options are possible (based on aspect, slope, and field shape). A dark grey Conditional Node included inside the module Activity Node represents the required exclusivity of a choice that is available during the iteration. *Trajectory Calculation* is the core module, which creates the optimized (modeled) trajectory. The whole module is encapsulated inside the light grey expansion region, whose presence indicates the iterative character of the process. The logical structure of the module itself is expressed using a white Activity node to enhance readability. The module *Bypass The Obstacles* adjusts the previously calculated trajectory to avoid possible obstacles.

### 4.2. Expression of the Developed Algorithm Through Pseudocode

Pseudocode is divided into identical building blocks, as defined in methodology (Section 3.4). The resulting pseudo-code originates from Structured Basic style pseudocode combined with notes (comments) expressed in natural language. An example of the developed pseudocode is depicted in Alghoritm 1, while the full pseudocode is available in Supplementary Materials.
**Alghoritm 1.** A portion of pseudo-code for an optimized (modeled) farm machinery route algorithm (a function of the splitting of a field of complex shape into segments).*function splitVertex* *{* *boundaries.vertex* *identify.vertex* *for i, i = 0, i++* *calculate angle between vertex i, i + 1 and i + 2* *if angle > 180* *{*   *perpendicular.cut*  *create new boundaries*  *splitVertex* *}*  *else* *i++* *}*

### 4.3. Comparison of Reference (Real) and Optimized (Modeled) Trajectories

**Figure 7:**
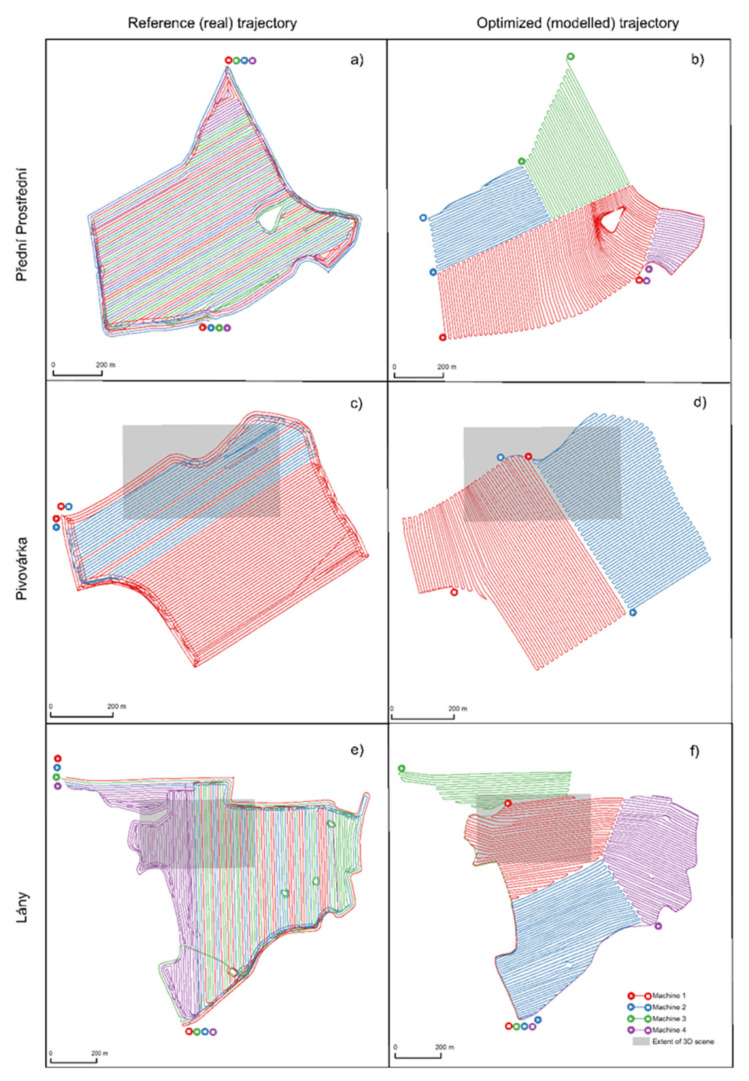
Map-based presentation of reference (real) trajectories measured at Rostěnice Farm (**a**,**c**,**e**) and optimized (modeled) trajectories with an identical number of harvesting sequences (**b**,**d**,**f**).

A comparison of the two following kinds of trajectories is presented in Figure 7:

- Manually created trajectories (similarly to reference—real—trajectories, as described in Section 3.2) according to the developed pseudo-code, and;
- Pre-processed real farm machinery trajectories for three fields at Rostěnice Farm.

The spatial pattern is the most tangible difference. In reality, more machines were employed in the adjacent lines, while the optimized trajectories divided a plot into segments that were intended to be operated by one machine. Another difference lies in the locations of entry/exit points. More entry/exit points are available in the optimized trajectories. All of the optimized entry/exit points were compared to real conditions, i.e., whether they were places at which a machine could safely enter or exit a plot in reality (see Figure 7).

A summary of the results of the descriptive statistics is provided in Table 2, while full statistical data are provided in Supplementary Materials. The following major differences between reference (real) and optimized (modeled) farm machinery trajectories were identified.

Table 3 depicts an overview of ranges of differences between reference (real) and optimized (modeled) trajectory lengths, elevation gains and numbers of turns. The following statements can be made on the basis of Table 2 and Table 3. The optimized (modeled) routes of farm machines developed in line with the presented algorithm were about 14% shorter than the (real) reference farm machinery trajectories. When taking into account an identical number of harvest sequences, the length savings varied between 10% (Přední Prostřední field) and 17% (Lány field). It should be noted that none of the optimized (modeled) trajectories were longer than the reference (real) trajectory (see Supplementary Materials for all the details).

In contrast, the number of turns in optimized (modeled) routes was around 40% higher than in the case of reference (real) farm machinery trajectories. When taking into account an identical number of harvest sequences, the increase in the number of turns varied between 11% (Lány field) and 81% (Přední Prostřední field). The increase in the number of turns results from the two following factors. First, plot segmentation results in the need for turns within the plot itself (see Figure 7, part f, the Lány field). Second, the longest edge of a plot is not followed as a default basis for optimized trajectories (due to the condition of in-contour farming), in contrast to agronomic practice.

**Figure 8:**
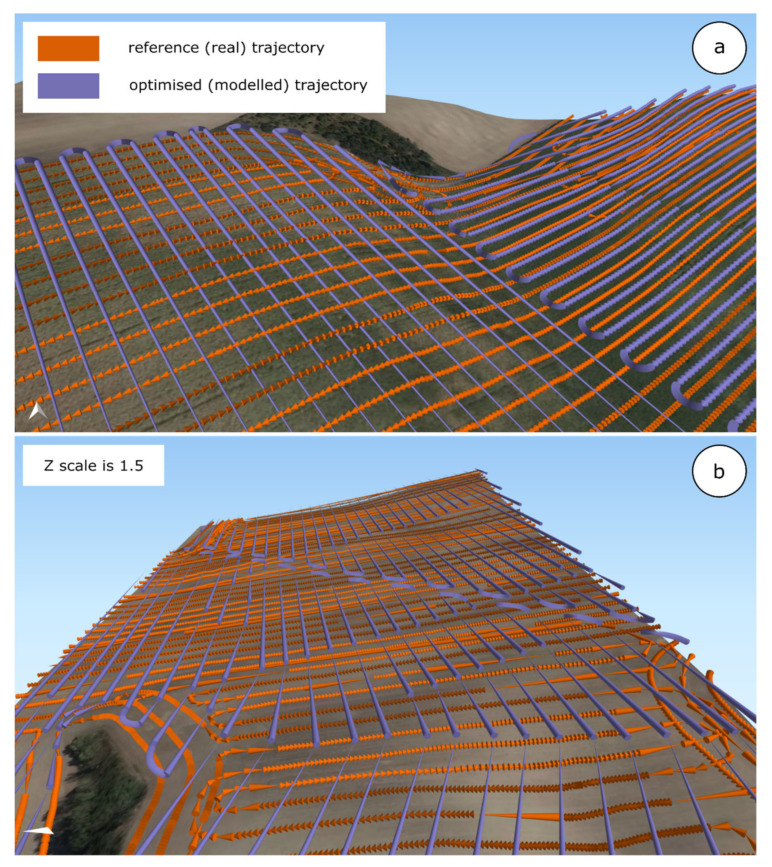
Three-dimensional visualization of reference (real) trajectories and optimized (modeled) trajectories with an identical number of harvesting sequences—from Pivovárka field (**a**) and Lány field (**b**) at Rostěnice Farm, Czech Republic.

Elevation gains (see also Figure 8) were on average 29% smaller for optimized (modeled) farm machinery routes in comparison to reference (real) trajectories. Savings on elevation gain varied from 14% (Pivovárka plot) to 53% (Přední Prostřední plot). The a priori impact of trajectory optimization was confirmed: in-contour farming minimizes elevation gain (as defined in Section 3.3).

The optimization of field entry and exit points according to their altitude had the following impact in the conducted experiment. The inclusion of field entry/exit points in trajectory planning increased the length of the optimized trajectories by an average of 4%. This average value varied from 1% (Pivovárka plot) to 8% (Lány field). This variance results from:

- The size of the plot: the larger the plot, the longer the trajectory;
- The shape of the plot: the more irregular the shape is, the longer the trajectory;
- The number of harvesting (or, in general, machine) sequences: the higher the number of plot segments is, the longer the trajectory.

The optimization of field entry/exit points also had an impact on the elevation gain. The inclusion of field entry/exit points in trajectory planning increased the elevation gain of the optimized trajectories by an average of 7%. Such an average value varied from 2% (Pivovárka plot) to 14% (Lány field). Relative height variation was the most important driver of elevation gain with respect to the elevation-based entry/exit points.

The optimization of field entry/exit points had no impact on the number of turns.

## 5. Discussion

### 5.1. Input Requirements for Designing a CTF-Based Algorithm

The development of an algorithm to optimize (modeled) (farm) machinery planning seems to be an approach addressed by several scientific domains. The following discussion highlights the input requirements that are raised across all the domains:

- The majority of related work addresses the development of an algorithm for optimizing (modeled) farm machinery trajectories in only two dimensions. Three-dimensional-based approaches are rare and were only described by Hameed [7] and Jin and Tang [10]. Indeed, 3D-based plot segmentation and the optimization of entry/exit points were not identified at all in the state-of-the-art analysis.
- Turns seem to be described in detail in most related work, with the most complex approach provided by Jin and Tang [18]. The spatial pattern of the developed trajectories follows a simple schema of turns connecting two adjacent lines of the trajectory [8].
- A constant radius of turn was employed in the current study. This constant radius was equal to the parameters of the harvesters used at Rostěnice Farm. In contrast, none of the analyzed papers addressed the challenge of a variable machine radius. As such, it remains open for further research.
- Obstacles are mainly modeled theoretically, as described, for example, in Yan, Wang and Chen [16]. The research presented in this paper deals with real long-term obstacles (e.g., power poles). These were a part of the data adopted from Rostěnice Farm and identified as permanent barriers.
- Related work mostly presents one machine operating on one plot. The harvesting time for a whole plot within the conducted study varied in reality from 8.5 to 17.5 h. More harvesters were assumed in the presented research; we can therefore talk about “harvester-days”.
- Entry and exit points were defined a priori in all the analyzed papers. In contrast, the presented research aimed at the optimization of entry and exit points. The procedure is described in Section 3.4., item D.a.ii. Agronomic practices during the conducted experiment did not follow this approach, as demonstrated in the reference (real) trajectories. As a consequence, the resulting real trajectory had to be extended for a significant part of the plot boundary at the beginning or at the end of the conducted operation.
- The analyzed papers did not address the return from a (finished) plot segment to a place that—in reality—allowed the given machine to truly leave the plot. Trajectory length, number of turns, as well as elevation between the start and end of a plot segment on the one hand and the entry/exit points of a given plot on the other were added to statistical evaluations. Such an approach enhances the work of Kroulík et al. [24].

In summary, input requirements for agronomic practice need to be variable. An algorithm which is successful in practice needs to be parametric to satisfy the ad hoc priorities of agronomists relating to current weather, type of crop, etc. It should also be emphasized that the research presented in this paper focused only on cereals. Other crops can have additional or different input requirements.

### 5.2. Ways to Formalize the Identified Requirements

Three major approaches can be identified with respect to formalizing the requirements for developing an algorithm for optimizing (modeled) machinery planning:

- The use of mathematical expressions that are most commonly used to describe a particular issue in detail. A typical example can be found in Jin and Tang [18], who defined various kinds of turns.
- The use of flow diagrams, as presented by Hameed [7] and Hameed et al. [21]. These papers formalize the developed algorithm also through a set of UML sequence (flow) diagrams as a general-purpose, developmental, modeling (graphical) language in the field of software engineering.
- The employment of pseudocode, as presented by Driscoll [6], Hameed [7] and Hameed et al. [21]. Pseudocode is commonly used as a basis for implementation.

These ways of formalizing approaches are the basis, among others, for (1) the automatization of the trajectory optimization process, (2) (semi)autonomous driving, and (3) the computing of costs, as defined, e.g., by Lampridi et al. [4].

### 5.3. Differences Between Reference (Real) and Optimized (Modeled) Trajectories

Results concerning reference (real) trajectories on the one hand and optimized (modeled) trajectories on the other differed significantly according to the input requirements described in Section 5.1. The following statements provide a general summary of the results achieved within the conducted study:

- The definition of (1) turns and (2) connections between the start and end of a plot segment on the one hand and the entry/exit point of a given plot on the other are the key drivers that influence the evaluation of an optimized (modeled) trajectory. Both aspects defined here significantly influence the length of a trajectory and the number of turns.
- Three-dimensional-based modeling does not have a significant influence on the length of trajectories in contrast to the previous point. However, a 3D-based approach was followed in the conducted experiment primarily to address soil erosion. Proper evaluation of erosion-related consequences would require an erosion model. The complexity of such research was beyond the scope of this paper; for details, see, e.g., Chyba et al. [9].
- Input requirements are, in reality, usually set in accordance with the ad hoc decisions of agronomists, who take into account, e.g., crop type. Such a fact emphasizes the need for a parametric and scalable model, as noted in Section 5.1.
- Different configurations of farm machinery were only partly verified in the conducted study, using two span widths in accordance with two vehicles that operate in reality at Rostěnice Farm.

The evaluation of soil compaction risk was performed in the conducted experiment only on the simple basis of the resulting length of a trajectory. The shorter the trajectory is, the lower the risk of soil compaction is. A full expression of the relationship between trajectory and compaction is beyond the scope of this paper, e.g., see Chyba et al. [9].

The evaluation of fuel consumption as well as of machinery wear also remain future tasks because of their complexity on the one hand and the given scope of this paper on the other. Fuel consumption within the defined scope is being addressed by Driscoll [6], Hameed [7], or Rodias et al. [8].

## 6. Conclusions

The presented research provides an open, modular, and scalable proof-of-concept solution for an advanced optimized (modeled) farm machinery route algorithm. The developed algorithm is a parametrical one with optional features. *Plot fragmentation* is one such feature, as this module can be deactivated if desired.

The outcomes were formally documented through a UML sequence diagram as well as through structured basic style pseudocode incorporating notes (comments) expressed in natural language descriptions. The algorithm in its current phase of development is intended for trajectory planning. In contrast to similar work, the presented development also addresses (1) the division of a plot into segments that may operate more machines simultaneously, (2) the full-employment of elevation, and (3) the optimization of the entry and exit points of plot segments.

The primary motivation for developing an optimized (modeled) farm machinery route algorithm was to minimize the risk of soil compaction. The evaluation of soil compaction within the study was conducted only indirectly, through shortening the length of the trajectories on three plots. Optimized (modeled) farm machinery routes were compared to reference (real) trajectories at the fully operational Rostěnice Farm in the Czech Republic. The optimized trajectories were in general about 10% to 17% shorter than their reference (real) counterparts. Elevation gain was reduced by an average of 29% in the conducted study. Both aspects indirectly imply fuel savings; however, exact verification is the subject of ongoing work. In contrast, the number of required turns rose by an average of about 40%.

The number of turns has a huge impact on the economic efficiency (in terms of fuel consumption and time) of the developed algorithm. It remains an open question whether fuel and time savings with respect to trajectory length and elevation gain can outweigh the negative economic impacts resulting from a significant increase in the number of turns. A broader picture needs to combine both economic and ecological points of view. For example, economic benefits need to be weighed against the risk of increased soil compaction.

More detailed evaluations would require the employment of an erosion model and a model for fuel consumption. Both aspects were, due to their complexity, beyond the scope of the presented research and remain topics for future research. Future work should also aim at increasing machine efficiency, determined by combining trajectory length with the number of turns.

## Figures and Tables

**Table 1:** Details of (real) reference farm trajectories measured at Rostěnice Farm.

Name	Date of Harvest	Number of Harvest Sequences	Number of Harvesters	Number of Measurements	Area (ha)	Number of Turns
Pivovárka	19.9.2018	2	1	26,655	46.0	93
Lány	14.7.2017	4	3	37,115	70.4	136
Přední Prostřední	14.7.2017	4	4	25,580	64.3	98

**Table 2:** Descriptive statistics: comparison of trajectory length, elevation gain, number of turns (headlands) for reference (real) trajectories and optimized (modeled) trajectories. Note, full statistical details are provided in Supplementary Materials.

Plot	Trajectory	Length		Turns		Elevation	
		Length [m]	Difference [%]	Number	Difference [%]	Gain [m]	Difference [%]
Pivovárka	Real	59762	N/A	93	N/A	2075	N/A
	Optimized-2 sequences	51012	−14.6	121	30.1	1784	−14.0
Přední Prostřední	Real	73430	N/A	98	N/A	2862	N/A
	Optimized- 4 sequences Option 1	66051	−10.1	177	80.6	1352	−52.8
Lány	Real	87614	N/A	136	N/A	1865	N/A
	Optimized- 4 sequences Option 1	72300	−17.5	151	11.0	1510	−19.1

**Table 3:** Descriptive statistics: range of differences in trajectory length, elevation gain, and number of turns (headlands) between all optimized (modeled) trajectories and reference (real) trajectories.

	Length Difference [%]	Turns Difference [%]	Elevation Difference [%]
Max	−20.3	103.1	−54.1
Min	−10.1	11.0	−12.3
Mean	−15.6	50.2	−25.6
Std deviation	3.5	34.4	17.9

## Data Availability

The datasets generated during and/or analyzed during the current study are available from the corresponding author on reasonable request.

## Supplementary Materials

The following are available online at https://www.mdpi.com/article/10.3390/s21092980/s1, Figure S1: UML activity diagram for a “*Bypass the obstacles*” module; Figure S2: UML activity diagram for a “*Plot fragmentation module*”, “*Division**_Based_On_Vertices*” function, respectively; Figure S3: UML activity diagram for a “*Plot fragmentation module*”, “*Division_Based_On_Aspect*” function, respectively; Figure S4: UML activity diagram for a *“Plot fragmentation module”*, *“Division_Based_On_Slope”* function, respectively; Figure S5: UML activity diagram for a *“Trajectory calculation*” module, *“Initiation-submodule*”, *“Calculate_Ideal_Move_Vector”* function, respectively; Figure S6: UML activity diagram for a *“Trajectory calculation”* module, rotation’ function, respectively; Figure S7: Map-based presentation of all options of optimized (modeled) trajectories according to the number of harvesting sequences; Table S1: Descriptive statistics: a statistical comparison of all trajectories’ length, elevation gain, number of U-turns (headlands). Note the table below is an extended version of Table 2 as presented in the paper; Table S2: Descriptive statistics: a statistical comparison of differences between all trajectories’ length, elevation gain, number of U-turns (headlands) when taking into account entry/exit points; Complete pseudocode.
