# Supplementary material for: Towards the Development and Verification of a 3D-Based Advanced Optimized Farm Machinery Trajectory Algorithm
Source: Sensors (Basel). 2021 Apr 23;21(9):2980. doi: 10.3390/s21092980 (PMC8123056; doi:10.3390/s21092980)
Supplement: Supplementary file 1 [file sensors-21-02980-s001.zip › sensors-1161762-SI.pdf]

sensors

MDPI

Supplementary materials:

1. UML activity diagrams

Note an overall UML activity diagram documenting the developed algorithm is depicted in Figure 5. Supplementary Materials contain seven remaining UML diagrams to demonstrate a more detailed level of the developed algorithm

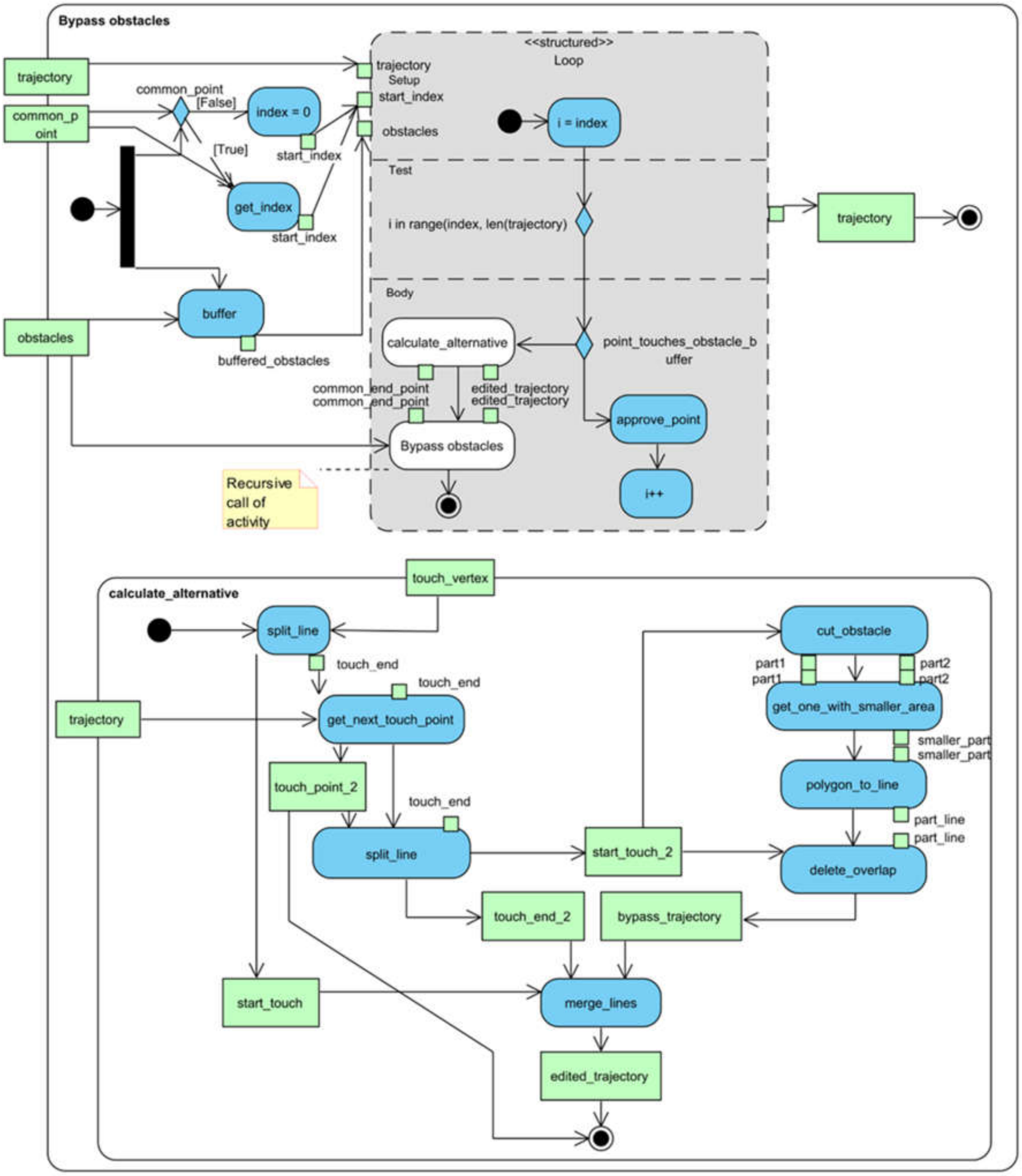

Figure S1. UML activity diagram for a '*Bypass the obstacles*' module.

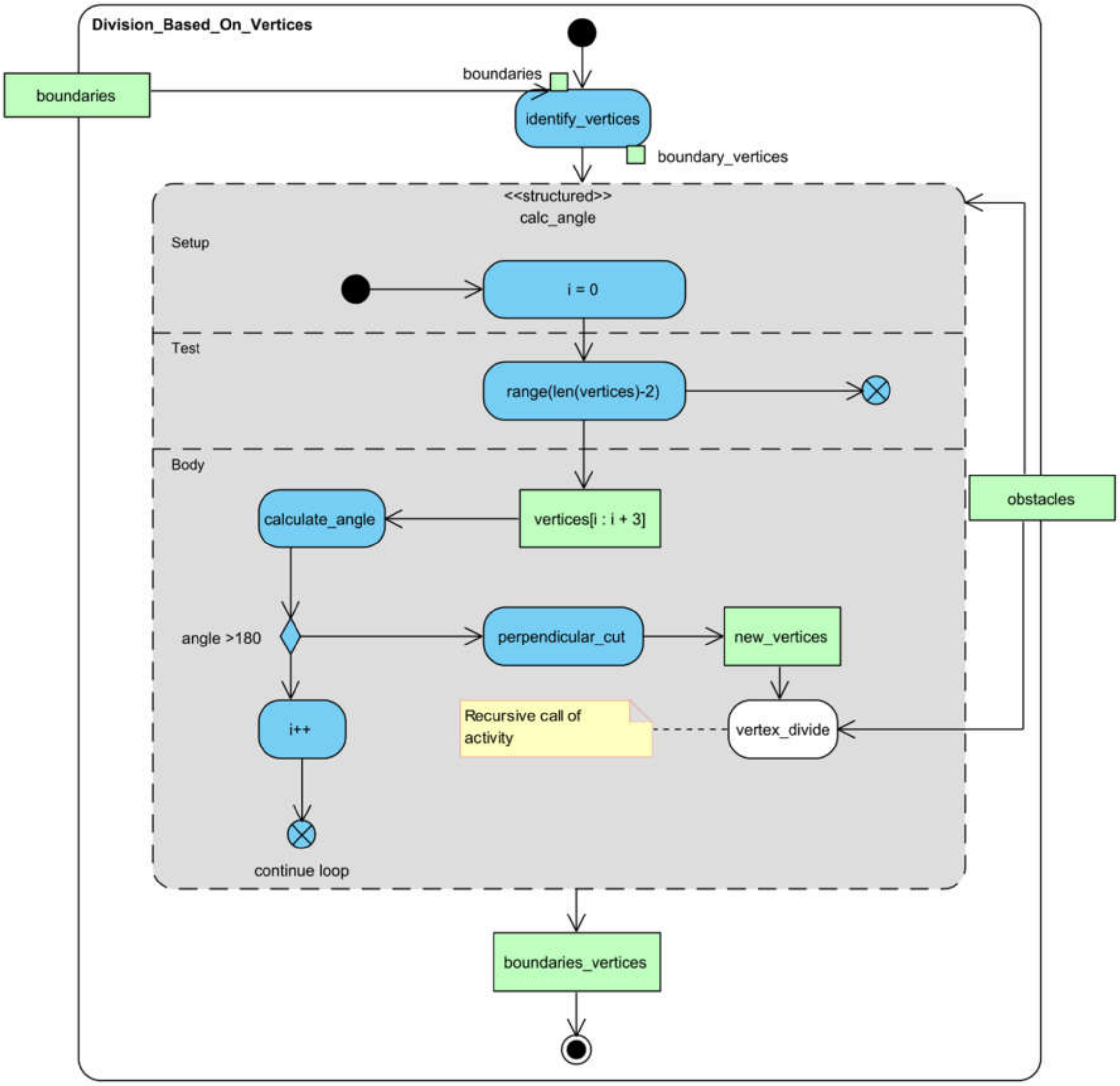

Figure S2. UML activity diagram for a '*Plot fragmentation module*', '*Division_Based_On_Vertices*' function respectively.

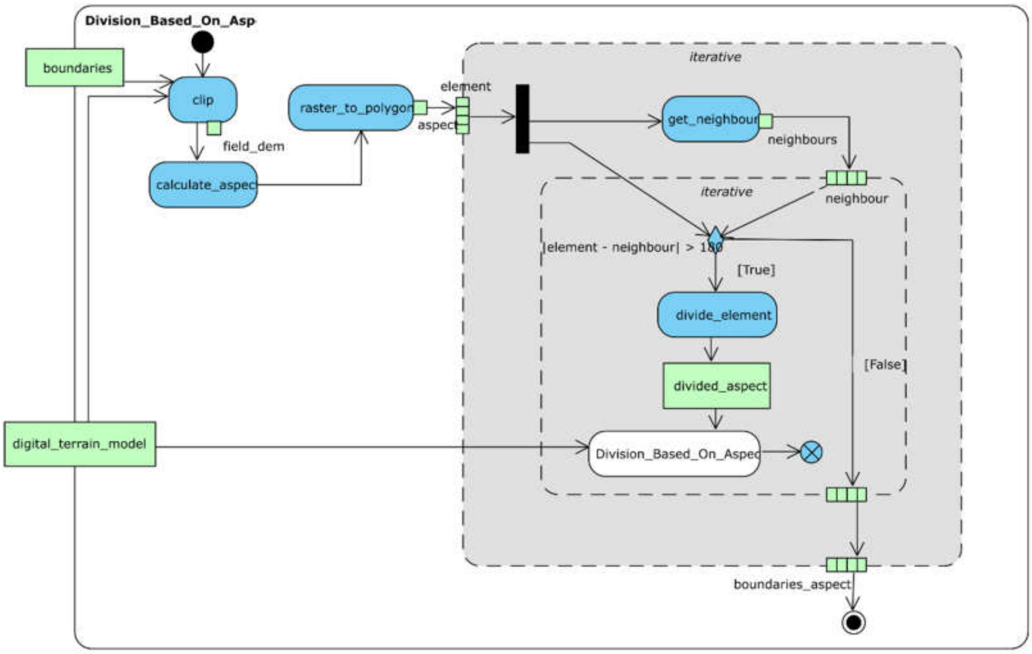

Figure S3. UML activity diagram for a '*Plot fragmentation module*', '*Division_Based_On_Aspect*' function respectively.

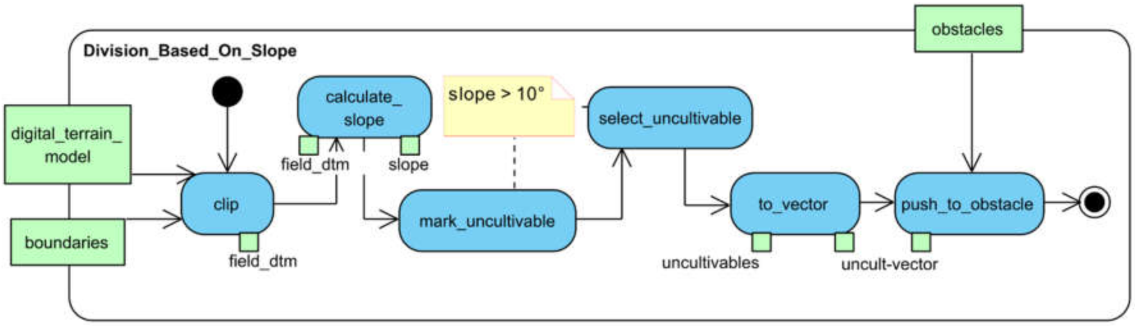

Figure S4. UML activity diagram for a '*Plot fragmentation module*', '*Division_Based_On_Slope*' function respectively.

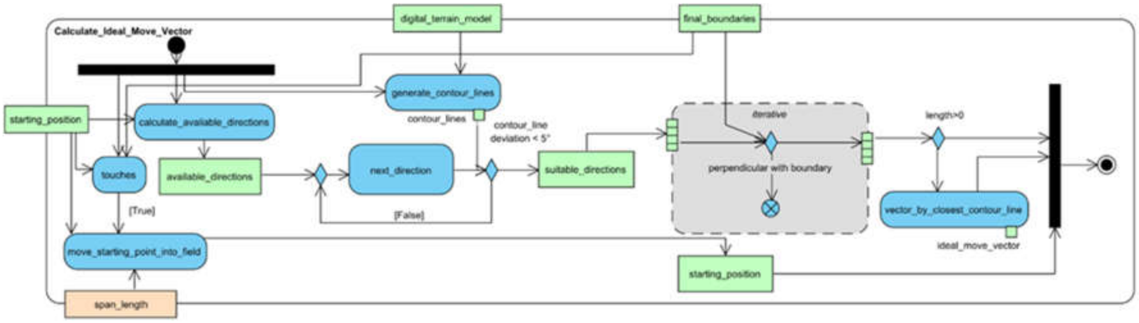

Figure S5. UML activity diagram for a 'Trajectory calculation' module', 'Initiation-submodule', 'Calculate_Ideal_Move_Vector' function respectively.

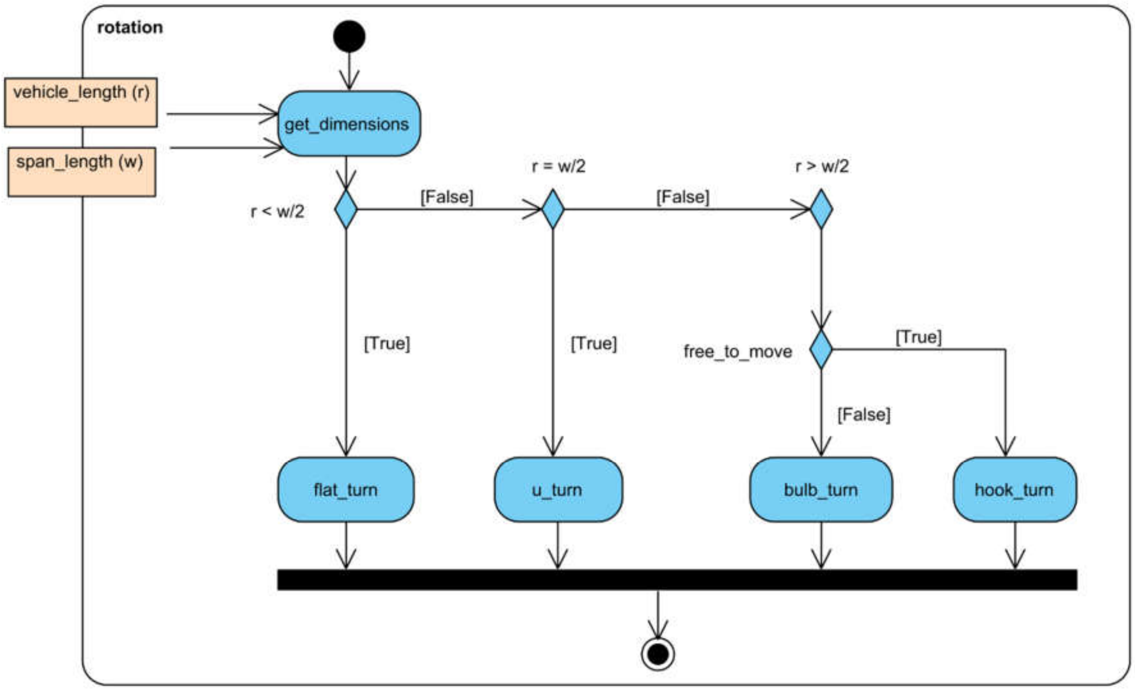

Figure S6. UML activity diagram for a '*Trajectory calculation*' module, rotation' function respectively.

2. Complete Pseudocode

Algorithm for optimal planning trajectories

```
main                    //function calls
{
    findData
    boundary
    obstacles
    splitVertex
    exposition
    slopeDivide
    rotation
    findStart
    checkTrajectory
    calculateIdealMoveVector
    bypassObstacles
    end
}

Vehicle length = r
span lenght = w


function findData           //put every data for calculation - elevation model, boundaries e.a.
{
    ask for DATA
}

function boundary           //function for checking boundaries, find correct format
{
    detect geometry
        if geometry is polygon
{
   we have boundaries
}

        else if geometry is polygon
{
  calculate boundaries from DATA
}

        else
            ERROR
}

function obstacles    //for example, if you have some organic obstacle that is active only during the growing season
{
    ask in attribute table on obstacle
    if yes
    {
        create buffer and say this is error place
    }
}
```

```
function splitVertex                    // you could choose this function, real calculation is in 2D
{
    boundaries.vertex
    identify.vertex
    for i, i=0, i++
{
                calculate angle between vertex i, i+1 and i+2
    if angle>180
    {
                                    perpendicular.cut
                                    create new boundaries
                                    splitVertex
    }
                    else i++
}
}

function exposition                     // you could choose this function, identifies natural changes in the landscape
{
    aspect.ELEVATIONMODEL
    compare angle in line z with neighbours
    if   i>180
        split by boundary
}

function slopeDivide                    // identification obstacles from nature
{
    add ELEVATIONMODEL
    clip.ELEVATIONMODEL by boundaries
    for i, i=0, i++
    {
    calculate.slope = ELEVATIONMODEL
    }
    for i, i=0, i++
                {
                    if slope >=10
                    push to obstacles
                }

}

function rotation                       // definition of shape of rotation way by Jin and Tang 2010
{
    if   r < w/2
        choose Flat turn
            elseif r = w/2
                choose U turn
    elseif r > w/2
                if one part is limited
                    If TRUE
                        choose hook turn
                    elseif FALSE
                        choose bulb turn
}
```

```
function findStart                              // opportunity for start
{
      find highest part of ELEVATIONMODEL
      check highest part != obstacle
      find nearest vertex
      nearest vertex = START
}

function calculateIdealMoveVector               // definition of trajectory calculus
{
   get DTM
   calculate contour lines
   get START position
   calculate a Variable directions
   for i, i=a Variable directions, i++
                      {
   if contour line devitation >5°
                          {
                          FALSE
                          start function again
      }
                         else TRUE
                         return this direction
                      }
}

function checkTrajectory                        // check of possibility completion of the task
{
      a.range
      if ourTrajectory>ad
      {
            split.to.half
      }

}

function bypassObstacles                        // Avoid the obstacles
{
     get trajectory
     fet obstacles
     if trajectory cross obstacles
     calculateAlternative
}

function calculateAlternative                   //how to avoid the obstacles
{
     get boundary
     get touch vertex
     split line
     bypass closer way
}

function end                                    // when the algorithm is over
{
```

```
        if every single piece of place are full
        STOP
        regressToEntry
    }

    function regressToEntry                    // go home function
    {
        find closest boundary
        find closest vertex on boundary of whole field
        for i=closest vertex, i>=0, i--
                {
                  calculate length from closest vertex on boundary to START
                return firstWay
                }
        for i=closest vertex, i<=last vertex, i++
                {
                calculate length from closest vertex on boundary to START
                return secondWay
                }
        if firstWay>secondWay
                {
                choose secondWay
                }
                else choose firstWay
}
```

3. All options of optimized trajectories in maps

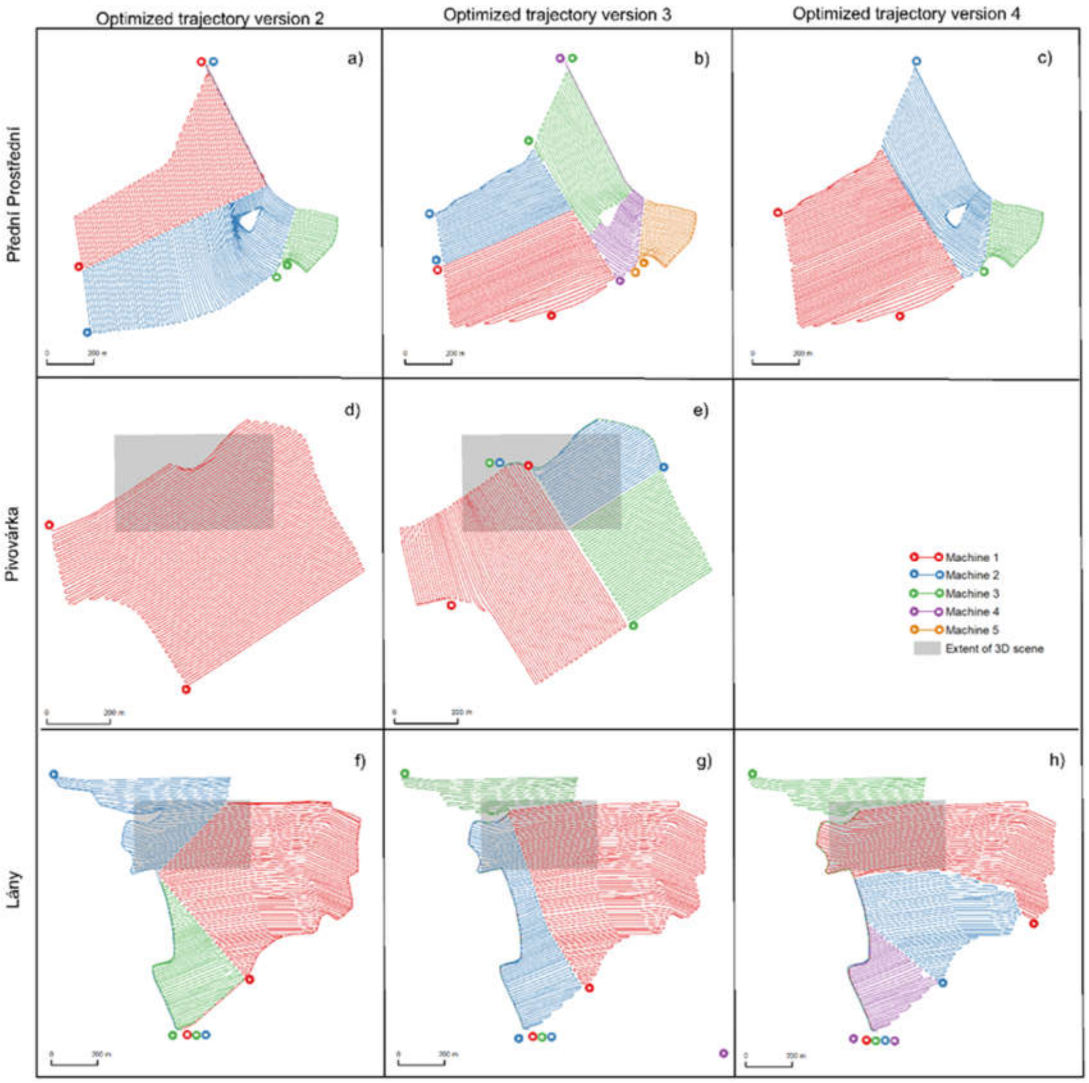

Figure S7. Map-based presentation of all options of optimized (modelled) trajectories according to the number of harvesting sequences.

4. Complete statistics

Table 1. Descriptive statistics: a statistical comparison of all trajectories length, elevation gain, number of U-turns (headlands). Note the table below is an extended version of Table 2 as presented in the paper.

Plot	Trajectory	Length		Turns		Elevation	
		Length [m]	Difference [%]	Number	Difference [%]	Gain	Difference [%]
Pivovárka	Real	59762.16	N/A	93	N/A	2074.79	N/A
	Optimized-1 sequence	50623.76	-15.29	121	30.11	1818.85	-12.34
	Optimized-2 sequences	51011.80	-14.64	136	N/A	1784.11	-14.01
Lány	Real	87614.32	N/A	151	11.03	1865.13	N/A
	Optimized-4 sequences Option 1	72299.52	-17.48	98	N/A	1509.51	-19.07
	Optimized-3 sequences Option 1	70924.94	-19.05	161	18.38	1499.34	-19.61
	Optimized-3 sequences Option 2	70770.85	-19.22	177	80.61	1512.03	-18.93
	Optimized-4 sequences Option 2	69841.97	-20.28	40.58	N/A	1461.37	-21.65
Přední Prostřední	Real	73429.95	N/A	98	N/A	2862.37	N/A
	Optimized-4 sequences	66050.45	-10.05	177	80.61	1351.52	-52.78
	Optimized-3 sequences Option 1	62588.99	-14.76	122	24.49	1342.00	-53.12
	Optimized-5 sequences	62390.99	-15.03	150	53.06	1314.00	-54.09
	Optimized-3 sequences Option 2	65761.20	-10.44	199	103.06	1534.16	-46.40

Table 2. Descriptive statistics: a statistical comparison of differences between all trajectories length, elevation gain, number of U-turns (headlands) when taking into account entry/exit points.

Plot	Trajectory	Length		Turns		Elevation	
		Length [m]	Difference [%]	Number	Difference [%]	Gain	Difference [%]
Pivovárka	Real	N/A	N/A	N/A	N/A	N/A	N/A
	Optimized-1 sequence	0	0	0	0	0	0
	Optimized-2 sequences	423.65	0.71	0	0	32.26	1.55
Lány	Real	N/A	N/A	N/A	N/A	N/A	N/A
	Optimized-4 sequences Option 1	6782.37	7.74	0	0	244.85	13.13
	Optimized-3 sequences Option 1	6131.66	7	0	0	260.34	13.96
	Optimized-3 sequences Option 2	5474.46	6.25	0	0	234.97	12.6
	Optimized-4 sequences Option 2	5238.33	5.98	0	0	208.57	11.18
Přední Prostřední	Real	N/A	N/A	N/A	N/A	N/A	N/A
	Optimized-4 sequences	3184.34	4.34	0	0	109.06	3.81
	Optimized-3 sequences Option 1	2944.61	4.01	0	0	100.63	3.52
	Optimized-5 sequences	2589.74	3.53	0	0	106.59	3.72
	Optimized-3 sequences Option 2	1352.81	1.84	0	0	89.04	3.11
